# Development and testing of the Hereditary Diseases and Genetic Testing (HD-GT) scale

**DOI:** 10.1186/1897-4287-9-S1-O3

**Published:** 2011-03-10

**Authors:** Christine Way, Kathy Watkins, Holly LeDrew, Valerie Ludlow, Mary Jane Esplen, Deborah Gregory, Patrick Parfrey

**Affiliations:** 1 School of Nursing, Memorial University of Newfoundland, St. John’s, NL, Canada; 2 Centre for Nursing Studies, Eastern Regional Integrated Health Authority, St. John’s, NL, Canada; 3 Clinical Epidemiology Unit, Faculty of Medicine, Memorial University of Newfoundland, St. John’s, NL, Canada; 4 Department of Psychiatry, Faculty of Medicine, University of Toronto, Toronto, ON, Canada; 5 Eastern Regional Integrated Health Authority, St. John’s, NL, Canada

## Background

Despite the expanding research base on the genetic testing process, limited insight exists on how personal understandings of hereditary cancer as well as situational and contextual factors influence an individual’s decision-making prior to and following predictive testing for hereditary non-polyposis colorectal cancer (HNPCC). The failure of existing scales to detect psychosocial and behavioral difficulties in this population has led researchers to question the adequate sensitivity of these instruments. The purpose of this study was to develop and validate measures for evaluating the preparedness for and experiences during and following predictive testing for HNPCC.

## Methods

A grounded theory study of individuals from families with a confirmed HNPCC presence provided the data for item generation and scale construction. The *Hereditary Diseases and Genetic Testing* (*HD-GT*) scale consists of 8 scales designed to measure acceptance of hereditary-based diseases and engagement in the genetic testing process. Scale readability and content validity were assessed prior to data collection. The HD-GT scale was administered to a sample of individuals (N = 242; 141 carriers and 101 non-carriers) from families with a MSH2, MLH1 or MSH6 mutation. The Multitrait Analysis Program-Revised (MAP-R) evaluated data quality, and scoring and scaling assumptions.

## Results

Preliminary findings support the logic of the psychometric structure of the HD-GT. The rating scale steps (0 to 4) seem to have meaningful divisions as evidenced by the response spread. The MAP-R analysis supported item-to-scale appropriateness, scale reliability (≥ .75) and pattern of scale correlations (internal consistency scores significantly higher than intrascale correlations). Study findings suggest that the family history of cancer does have a significant impact on decision-making regarding genetic testing. There are also indications that individuals place high value on having all potentially at-risk family members participate in genetic testing, but are often challenged trying to convince them to accept the need for testing.

## Conclusions

The HD-GT scale represents an initial effort to evaluate personal, situational and contextual factors influencing genetic testing decision-making. Study findings suggest that the subscales appear to be sensitive enough to measure the wide-range of psychosocial and behavioral implications of genetic testing. Further, findings have important implications for health care providers. If providers are to work effectively with HNPCC families they must have the appropriate knowledge, education and skills to recognize the features of HNPCC, take thorough client and family histories, provide support and coordinate care for these individuals.

